# When Skin Damage Causes Death

**DOI:** 10.1371/journal.pbio.0060133

**Published:** 2008-05-27

**Authors:** Liza Gross

Our skin routinely shields us from microbes, allergens, and other environmental assaults, a yeoman's service we often take for granted—until that barrier is breached. In response to injury, be it a simple cut or a deep wound, keratinocytes, the cells that form the epidermal layer, proliferate and dispatch chemical messengers to enlist the healing services of immune cells. But new research shows that sometimes damaged skin can send the wrong message to its immune cell partners. Rather than recruiting immune cells to repair a wound, Raphael Kopan and colleagues report, defective skin can trigger a systemic, ultimately fatal immune response.

As a self-renewing tissue with four distinct layers of specialized keratinocytes, the epidermis must constantly generate new cells to replace the many thousands that die each day. Both rejuvenation and wound repair require the support of a new pool of specialized cells, a process that is mediated by the Notch signaling pathway, an ancient intercellular communication system found in most multicellular animals. To remain healthy, skin must maintain a delicate balance between cell differentiation and growth. Interfering with differentiation—by impairing the Notch pathway, for example—can cause serious defects in the epidermal barrier and lead to inflammatory skin disorders like psoriasis or atopic dermatitis, a type of eczema.

The source of new cells comes from the innermost, basal, skin layer, which harbors a population of stem cells that continually divide to generate the cells that will eventually populate the other layers. Notch appears to operate in one way to suppress basal cell proliferation and promote keratinocyte differentiation, while using a different mode to ensure a dynamic equilibrium between differentiation and growth.

**Figure:**
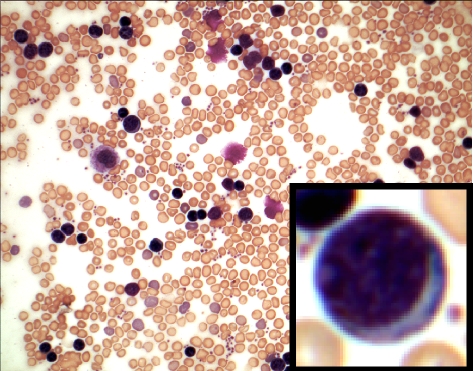
Dramatic expansion of pre- and immature B cells in peripheral blood is the hallmark of B-lymphoproliferative disorder, caused by defective skin differentiation seen in the newborn mice lacking Notch signaling in their skin. The inset shows a lymphoblast.

To investigate Notch's role in skin homeostasis and barrier formation, Kopan and colleagues used a protocol that removes multiple genes in the Notch pathway in keratinocytes during a narrow window of embryonic development in mice, when the blood system is forming. Most mice with no trace of Notch signaling in their skin developed chronic barrier formation defects akin to those seen in atopic dermatitis, and died about three weeks after birth. They also had extremely high levels of B cells, the white blood cells that produce antibodies to fight infection.

Paralleling the loss of Notch signaling, the researchers saw an increase in the expression of a cytokine called thymic stromal lymphopoietin (TSLP), in direct proportion to the severity of defects in cell differentiation and barrier formation. The spike in TSLP levels in turn triggered an abnormal expansion of pre- and immature B cells in peripheral tissues (B cell development in normal embryos is restricted to the fetal liver), leading to dangerously high levels of B cells, a condition known as B-lymphoproliferative disorder (B-LPD). In severe cases, the B cells ultimately infiltrate several organs in the mice, which results in death.

TSLP, which has been implicated in atopic dermatitis, can stimulate the development of the fetal pre-B cells (but not adult pre-B cells) in test tubes. This study confirms that exposure to high levels of TSLP during embryonic development can trigger a massive expansion of pre- and immature B cells, causing B-LPD and death. The finding that localized skin defects can cause a fatal systemic disease reveals a surprising, complex interaction between the immune system and the skin, and suggests many new questions to explore. Kopan and colleagues want to know, for example, how keratinocytes detect the severity of skin damage and translate this information into TSLP output. With a better understanding of the mechanisms underlying this skin–immune system connection, investigators may be able to develop new therapies to treat a wide range of incurable autoimmune-related skin disorders, including atopic dermatitis, an often debilitating condition that commonly affects children.

